# Electrospray mechanism for quantum dot thin-film formation using an electrohydrodynamic jet and light-emitting device application

**DOI:** 10.1038/s41598-020-67867-w

**Published:** 2020-07-06

**Authors:** Tuan Canh Nguyen, Woon-Seop Choi

**Affiliations:** School of Electronics and Display Engineering, Hoseo University, Asan, Chungnam 31499 Korea

**Keywords:** Materials science, Optics and photonics

## Abstract

A novel electrohydrodynamic (EHD) electrospray coating mechanism was proposed for the continuous fabrication of large-area quantum dot (QD) thin films for high-performance light-emitting diodes (LEDs). The size of QD droplets was systemically controlled using the stable EHD electrospray mode from a mixed solvent, which is a crucial factor for the formation of large and smooth QD thin films. The minimum amount of material consumption was achieved during the process by applying the unique coating system. A QD-LED device based on electrodeposited QDs showed a maximum luminance of 12,082 cd m^−2^, maximum current efficiency of nearly 4.0 cd A^−1^, and maximum EQE of 1.86%. This system demonstrates not only high reproducibility but could also pave the way for commercializing high-quality QD-LED devices.

## Introduction

Semiconductor nanocrystal quantum dots (QDs) have gained significant interest due to their unique properties, which include high color purity, high quantum efficiency, and tunable emission wavelength. They are considered to be alternative emissive materials for next-generation light-emitting diodes (LEDs) in lighting and display applications^1^. QD chemistry seems rather simple, but obtaining extremely small size distribution is an enormous challenge. The cost of QD production and device fabrication also needs to be minimized^2^.

QD materials are probably unsuitable for conventional vacuum evaporation deposition for fabricating optoelectronic devices like organic light-emitting diodes (OLEDs). Therefore, the development of an appropriate solution process is required. Solution processes with low cost have been developed for the QD layer, including spin-coating, transfer printing, direct contact printing, mist coating, and electrophoretic deposition^3,4^. Spin-coating is the most popular solution process for QD thin film formation in laboratories, but its application is limited in large-scale deposition applications. The relatively high cost of QDs and the loss of more than 96% of the solution during spin coating are strong reasons to search for an alternative deposition route that is capable of patterning and consuming a minimum amount of QD materials^5^.

In the electrohydrodynamic (EHD)-jet printing method, a liquid jet breaks up into fine droplets under the influence of an external electric field. There are various modes of the jet that is created from the QD solution, such as dripping, spindle, Taylor cone-jet, and multi-jet modes. Based on the individual purposes of fabricating electronic devices, an EHD printer can be used for continuous patterning, thin film deposition, and various printing. Most researchers are focusing on Taylor cone-jet mode to fabricate a matrix of dots^6^ or line patterns, and then depositing them on top of devices^7^. Cone-jet spray mode was successfully applied to deposit QD thin films, as an emitting material layer (EML) in the sandwich structure of QD-LEDs^8,9^. Even though the device performance was quite good, the root mean square (RMS) roughness was high due to the large QD droplets, and there were many droplet marks on the surface because of the quick solvent evaporation, which is still a challenge to be solved.

Electrospray mode was developed from an EHD-jet printing system as a potential solution process for the large-area fabrication, of smooth and thin QD films. It is relatively easy to control the process by controlling the applied power, flow rate, nozzle distance, spraying time, printing direction, and properties of the precursor solution. The EHD-jet printing process is a direct thin-film formation process that does not require pre-processing, and almost 90% of the QD solution is used. Therefore, the loss of QD materials can be significantly reduced by using an EHD-jet electrospray coating process compared to a simplified spin-coating solution process^10^.

Atomization is induced by an electric field between the solution and the collector, so the sprayed mist of QD particles has identical charges to self-disperse in the deposition space. As a result, the aggregation of QD nanomaterials before the deposition is minimized^11^. The deposition process is performed using a highly diluted solution of nanomaterials, so the dispersion of nanomaterials is easily flocculated in the solution, which should be improved considerably^12^.

In this study, we tried to solve these issues by controlling the solvent evaporation rate and reducing the size of sprayed droplets using EHD electrospray mode^13^ with a solvent mixture of QD solutions. The formation of a QD thin film is based on the ordering and collection of QD droplets by droplets. To understand the electrospray mechanism, the initial QD droplet size, solvent evaporation rate, and spraying time were estimated by applying a Scaling law and Rayleigh law to figure out the suitable EHD parameters and properties of the supply solution. We successfully developed an emissive QD layer by controlling the EHD parameters and treating the QD solution to obtain a homogeneous dispersion and maintain stable electrospray mode. A QD-LED with optimized electrosprayed QDs showed improved optoelectrical properties.

## Results and discussion

**Figure 1:**
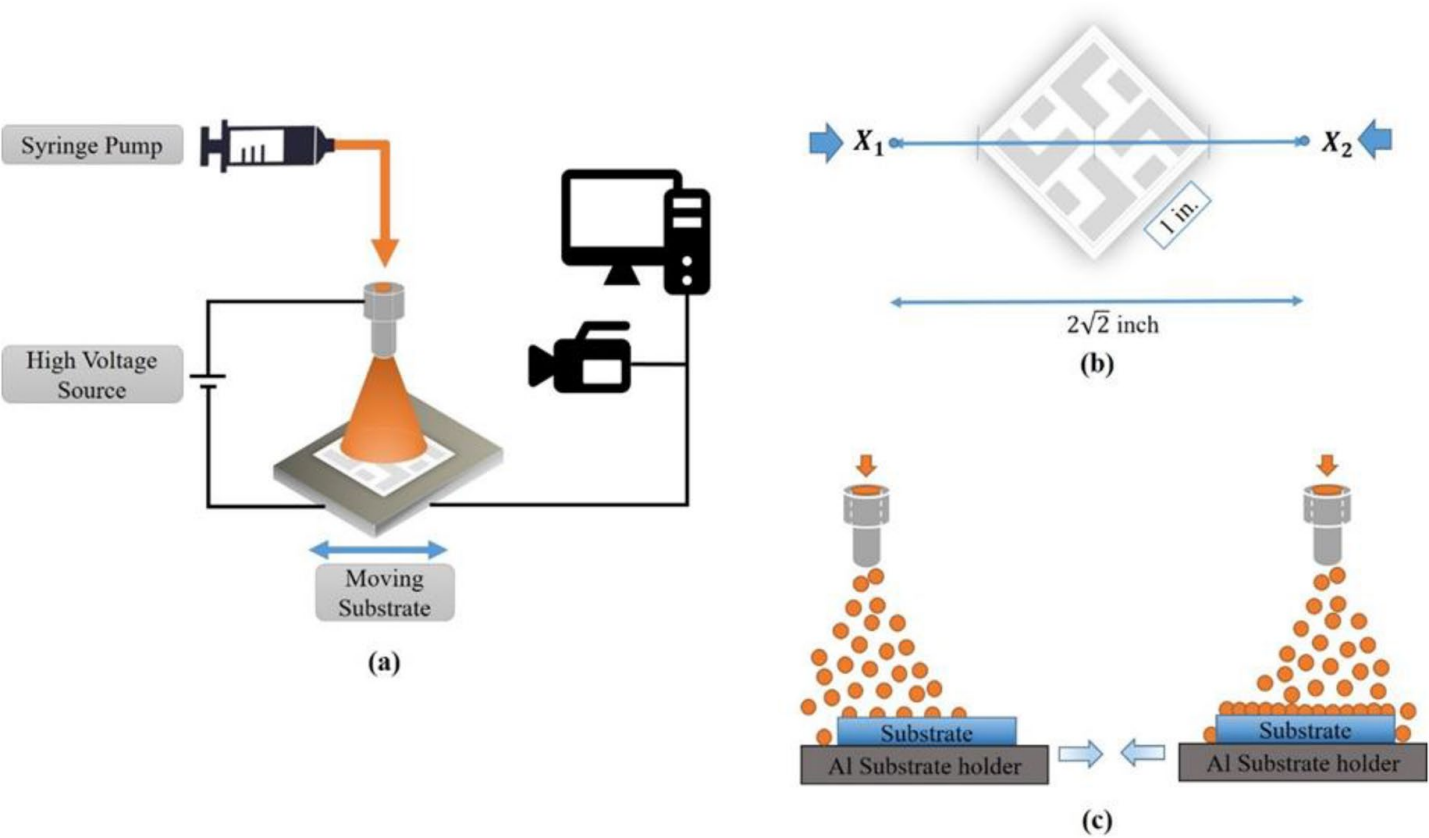
(**a**) Diagram of EHD electrospray coating systems, (**b**) moving direction of the stage, and (**c**) sequential electrospray coating process with two directional spray paths to avoid edge effect.

EHD electrospraying is a process where a liquid jet breaks up into fine droplets under the influence of an external electric field. The different printing jet modes in Fig. S1 in the Supplementary Information (S.I.) are determined by the competition of electric stress and the surface tension on the liquid–gas interface, as well as the kinetic energy of the liquid leaving the tip of the nozzle. Micro-nano aerosol droplets are generated by charging the liquid and flowing it through a capillary tube with an applied electric field as illustrated in Fig. 1a.

At low voltage, the surface tension of the liquid dominates the electric field, and liquid flows drop by drop at the capillary outlet. When the applied voltage increased, the electric field becomes stronger and elongates the liquid meniscus into the cone, multi-jet, and aerosol droplets (known as electrospray). The QD droplet size of the electrospray can be varied from a few hundred nanometers to micrometers depending on the process parameters for a given QD solution^14,15^. The electrospray mode formation is a complicated process that requires high applied voltage, low viscosity, and high working distance compared to other printing modes, as shown in Table 1.

**Table 1:** Various EHD printing modes with solution formulations and processing parameters.

EHD printing mode	Cone-jet mode	Multi-jet mode	Electrospray mode
Solution	QDs based hexane:*n*-hexane	QD based hexane:*n*-hexane:*n*-butanol	QD based hexane:*n*-hexane:*n*-butanol
Volume ratio	1:4	1:3.8:0.2	1:2.5:1.5
Applied voltage (kV)	< 4.7	7–8	8–10
Height of tip (cm)	1–5.5	4–6	5–6.5
Flow rate (µL/s)	0.008–0.128	0.032–0.064	0.016–0.064
Picture of EHD jetting modes	(a)	(b)	(c)

Images (a), (b), and (c) in Table 1 show the pictures of the cone-jet, multi-jet, and electrospray modes for three kinds of QD solution formulations. Under the applied voltage, the electric field penetrates into the liquid and leads to an enrichment near the meniscus. This causes a destabilization of the meniscus, the formation of a cone, and a jet charge (a) with an excess of positive ions. At higher voltages (> 4.7 kV), the Coulombic repulsion increases and destabilizes the initial QD droplets, which emit multi-jets (b). During the splitting of the initial QD droplet (the parent droplet), the evaporation of the solvent leads to repeated fission of the droplet^16^. The “progeny” QD droplets also evaporate and undergo fission. The steady electrospray mode formation (c) requires very high applied voltage (10 kV) and the suitable viscosity, surface tension, dielectric constant, and liquid electrical conductivity of the QD solution. In this mode, many meniscuses can be observed in multi-jet mode at the rim of tip, and become flat and gradually disappear. The steady electrospray mode can create a plume of QDs, which include many extremely tiny and invisible main jets with satellite jets.

QDs that are passivated by a ligand can be dispersed in solvents. Red QD-based hexane was mixed with n-hexane to obtain a lower concentration to generate fine droplets by electrospraying. However, the boiling point (b.p.) of n-hexane is 69 °C, which causes the solvent to evaporate quickly, resulting in high roughness of the droplet marks on QD films. The low viscosity of n-hexane also results in large initial droplets. Therefore, many solvents are being investigated to develop the electrospray mode and control the size of droplets, such as chloroform, ethanol, *n*-hexane, cyclohexane, cyclohexylbenzene, bicyclohexyl, and *n*-butanol.

Moreover, solvents should be carefully chosen to form a stable spray without dissolving the underlying layer. Unsuitable solvents may cause significant damage to the underlying organic HTL in QD-LED devices. Therefore, a mixed solvent of *n*-butanol (b.p. of 117.7 °C) with *n*-hexane was chosen for the QD solution to maintain a stable electrospray mode, a suitable rate of solvent evaporation, and uniform micro-nano-size of the droplets to obtain a wet continuous QD thin film with short deposition time. The solvents are completely compatible with QDs, and are not react with the trioctylphosphine (TOP) ligand of the surface core/shell QD nanoparticles.

The critical feature of the electrospray strategy is the self-dispersal of QD droplets into micro- to nano-size after electrospraying. Good dispersion results in a film with a uniform and smooth surface. Experiments have been performed using QD droplets generated at a potential of 10 kV to produce the smallest size of the droplets and obtain the highest quality of QD thin films. The estimated diameter of QD droplet marks on the ITO substrate is ~ 1 µm when using a typical flow rate of 0.016 µL/s and the tip height of 6.5 cm to make sure the mist covers all of the substrate. The QD thin film formation is a collection of progeny QD droplets. Therefore, the quality of the thin film highly depends on the size of the progeny QD droplets and the parent QD droplets.

**Figure 2:**
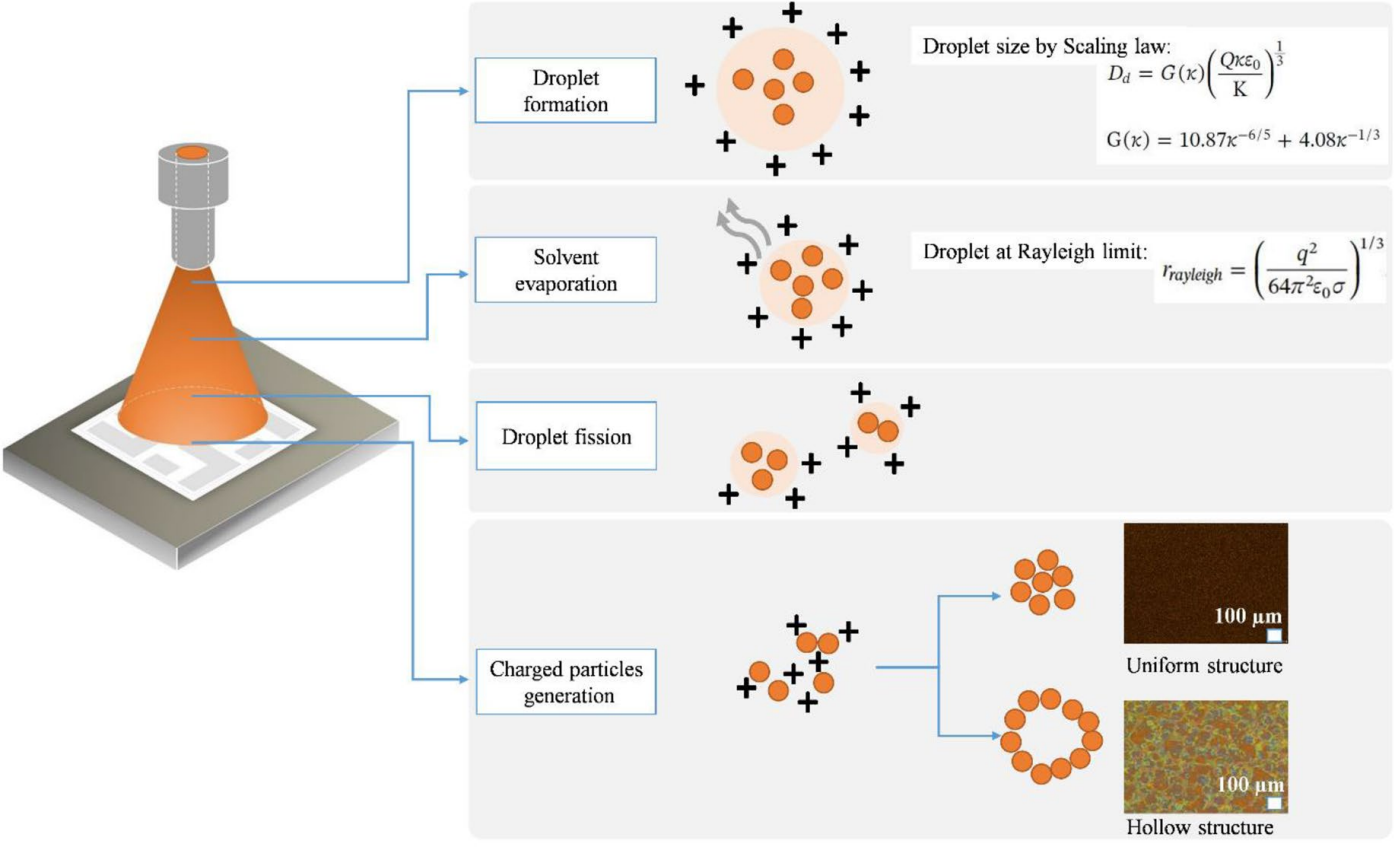
Mechanism of QD thin film formation by EHD electrospray.

To understand the mechanism of electrospray, it is first necessary to recognize the initial QD droplet size and the process of fission and evaporation, as shown in Fig. 2. Scaling law equation was used to calculate the diameter of the initial electrospray QD droplets^17^.

$$D_{d} = G\left( \kappa \right)\left( {\frac{{Q\kappa \varepsilon_{0} }}{K}} \right)^{\frac{1}{3}}$$

${D}_{d}$ is the droplet diameter (µm), $\kappa$ is the dielectric constant, Q is the flow rate, K is the liquid electrical conductivity (µS/cm), ${\varepsilon }_{0}$ is the permittivity of vacuum, and G($\kappa$) is shown in Eq. (1) in the S.I. This equation indicates that the QD droplet size can be controlled by changing the flow rate, relative permittivity, and liquid electrical conductivity of the supply solution.

Direct measurement of the QD droplet size is complicated, but the effect of this parameter on the QD droplet size is reflected in the morphology of the QD films. Because of this particularly mode structure, it is difficult to clarify how many jets in this plume of QD droplet. Therefore, to apply Scaling law and predict the diameter of initial QD droplets, we assumed that the flow is so divided among the number of jets, then the average flow rate per jet will be calculated by ${\left.\left(\frac{Q}{n}\right.\right)}^{1-\alpha }$, in which Q is the initial flow rate, n is the number of jets, α is coefficient. In case of the number of jets is small, we can predict the size of QD droplet by simulation, image analysis, or applying the Groove model. In spite of large number of jets in this research, we assume that there are hundred jets (n = 100) and α = 0 for simplification, then the average flow rate per jet will decrease 100 times.

The solution used in this study was a mixture of two solvents. When the solvent is *n*-hexane, the diameter of the initial electrospray QD droplets is estimated at about 0.68 µm. The diameter is reduced to 0.13 µm if the solvent is *n*-butanol. After being emitted from the needle tip, the mixture of *n*-hexane and *n*-butanol solvents in the QD droplets gradually evaporates, resulting in a significant increase of the charge density within the QD droplets. Rayleigh jets occur when smaller droplets are formed through constant charge fission, and then many progeny QD droplets with much smaller size are formed. The resulting progeny QD droplets undergo Coulomb fission again with continuous solvent evaporation until the QD droplets reach the collector. Only QD nanoparticles remain on the substrate with hemisphere or droplet shapes. The average diameter of this single island approximately from 500 nm to 2 µm was confirmed by SEM images, which is a bit bigger than the maximum calculated value of 0.68 µm.

During a QD droplet fall out of the tip, the diameter of individual QD droplet was gradually decreased due to solvent evaporation. This solvent evaporation process was also continuous on the partially wet QD droplet on the surface of the collector. The time of the solvent evaporation in the electrospray space can be evaluated by Eq. (2) in the S.I. The structure of the electrospray deposition layer can be investigated through the solidification of the QD droplets. The droplets touching the collector and the quick evaporation of the solvent can result in high roughness^9^. When bicyclohexyl (b.p. of 227 °C) is used as a co-solvent, the very high boiling point of the solvent leads to a wetting state of the thin film. The drying process at room temperature and the use of a hot plate result in the collection of rough concentric circle marks due to heterogeneous shrinkage on the surface, which is not suitable for sandwich structure devices. The mixed solvent of *n*-butanol and n-hexane showed a lower rate of solvent evaporation, which could provide better surface morphology.

It is essential to form continuous wet precursor films with QD droplets to achieve smooth and dense films without pin-holes and to minimize coffee ring effects. For this purpose, the droplets should remain fluidic on the substrate, to allow the QD droplets to merge and become part of a continuous wet film. Otherwise, they would dry prematurely, and the substrate would only collect QD nanoparticles, resulting in a porous and rough film.

With QD inks with n-hexane solvent only, QD droplets were jetted from the metallic tip, and the solvents were quickly evaporated^9^. The combination of n-hexane and n-butanol was chosen along with the printing parameters to make the flight time of the QD droplets shorter than the evaporation time of the solvents. The time of the solvent evaporation is also a factor that determines the parameters of the coating method. Moving the collector at 2 mm/s guarantees that the QD droplets deposit on the surface with uniform alignment (Fig. 1c). It also makes sure that the solvents completely evaporate in a moving step. However, the complicated drying process also depends on the flight time of the QD droplets through the tip and touching the collector.

The flight time of electrospray QD droplet between the needle and the substrate can be calculated as^17,18^.

$$\tau_{res} = \frac{{18\mu Qd^{2} }}{{ICD_{d}^{2} \Delta V}}$$

where $\mu$ is the viscosity of the fluid, d is the collection distance (6.5 cm), Q is the flow rate (0.016 µL/s), I is the droplet current, C is the Cunningham slip correction factor, and ${D}_{d}$ is the diameter of the droplet, as shown in the S.I. We estimated the flight time as between 0.23 and 0.85 ms, which correspond to solutions with *n*-hexane only and *n*-butanol only, respectively.

The collector was kept at 5–6 s after one moving turn (from X_1_ to X_2_, and repeat the movement), which is more than thousand times than the flight time of QD droplets to guarantee that all electrospray QD droplets are completely deposited on the collector. This intermission time also makes sure all deposited-QD droplets stay at a suitable wet-dry stage before being embraced by the next coating turn.

Besides the formation of electrospray mode, other primary factors were considered to achieve smooth QD thin films. Among those were the moving of holder substrate and the moving direction. The center of the ITO substrate was aligned along the centerline of the atomizer and tip of the nozzle to fit the center of the spray plume. The sketch of the cross-view in Fig. S3 shows the difference between the two kinds of arrangement substrates when they have moved along the side and the diagonal.

When the substrate was moved the side direction, the edge effect created a mound of QDs (Fig. S3a). Then, the next ZnO layer was spin-coated on a rough QD layer. The hydrodynamic effect from coating created many defects on the surface, resulting in low-performance devices with a little waste material. However, when the diagonal direction was applied, there was no substance mound on the edge. This was done primary for making smooth films by avoiding possible edge effects, resulting in device performance with material saving.

Due to the continuous movement, QD droplets are continuously coated onto the ITO substrate, and the thickness of the electrosprayed QD thin films can be easily and precisely controlled from 20 to 300 nm by changing the spraying time. However, the formation of a thin-film involves the alignment of nano-QDs droplet by droplet and layer by layer, so preparing QD films with over 300 nm thickness may be a limit for this electrospray method. Thus, this versatile EHD electrospray coating technique could be considered as a potential manufacturing process.

**Figure 3:**
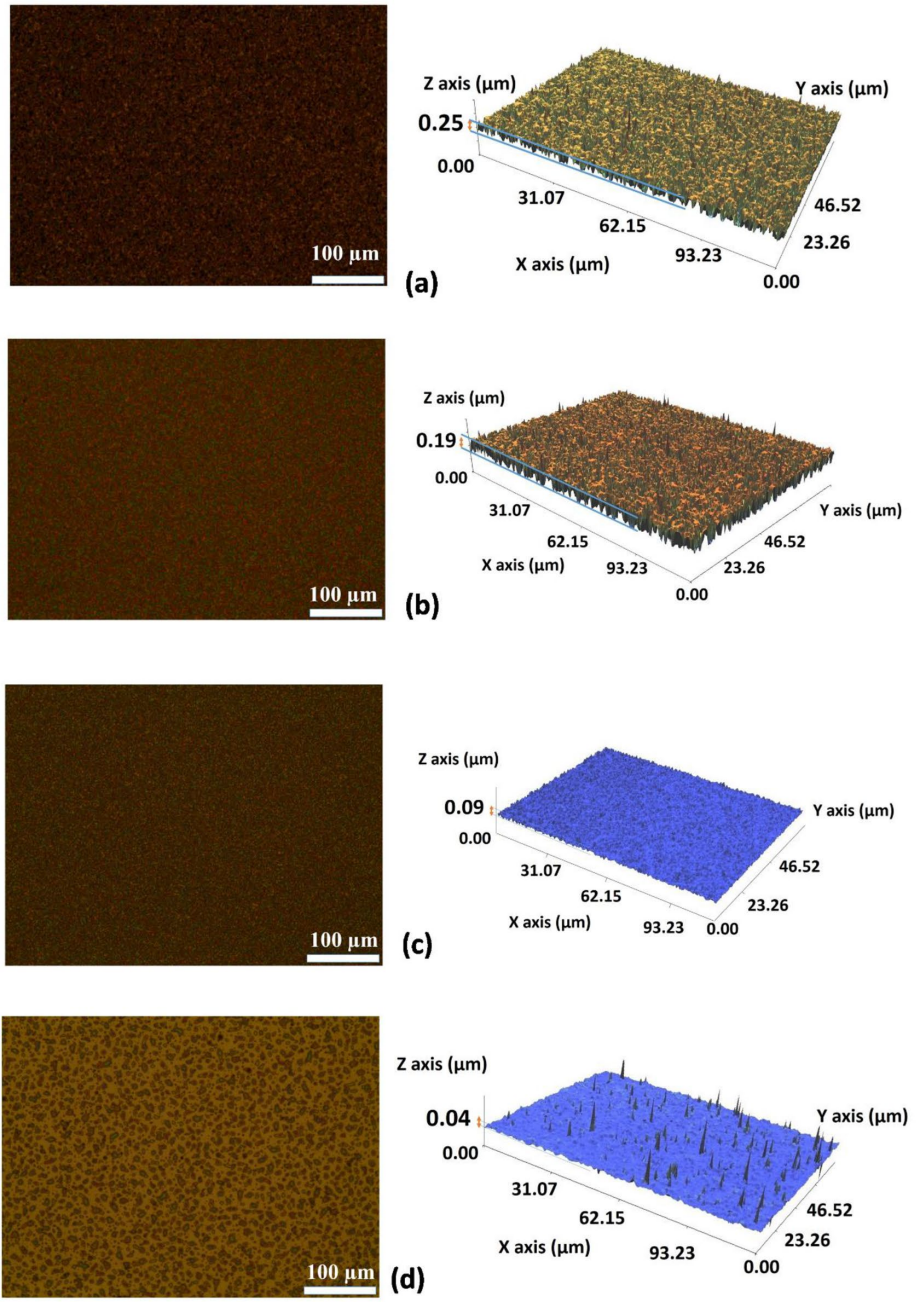
OM and 3D images of various electrosprayed QD thin films with (**a**) Solution A, (**b**) Solution B, (**c**) Solution C, and (**d**) Solution D.

Based on the above results, we analyzed the relationship among the EHD parameters, the effect of the solution on the printing mode of formation, the structure of QD droplets, and the morphology of QD films. Various films were prepared from four kinds of QD solutions as in Table 2. The surface morphologies are shown by OM images and corresponding NanoSystem WSI (White-Light Scanning Interferometry) 3D images in Fig. 3. With 15 min for electrospray coating, the OM images show a different structure on the film surfaces. The black dots in the OM image in Fig. 3a (Solution A) show separate grains and holes of dry QD particles. The correlating 3D images obtained by the NanoSystem are shown through assorted colors.

**Table 2:** Composition and viscosity of QD solutions.

Solution	Composition^a^ Red QD based-hexane:*n*-hexane:*n*-butanol	Viscosity (cP, estimated)
A	1:1:3	1.8
B	1:2:2	1.3
C	1:2.5:1.5	1.05
D	1:3:1	0.8

^a^Volume ratio.

The size and QD density were changed in Fig. 3c (Solution C), and the uniformity of the color shows that the surface becomes smoother and thinner. The green color of the 3D image in Fig. 3c and d also show the thickness of the electrosprayed QD thin film, which has much less roughness than those of Solution A and Solution B. With Solution C, the electrosprayed QD thin film shows the lowest roughness of 0.0308 µm and thickness of ~ 90 nm. At the same EHD conditions, the thickness of QD films obtained approximately 250 nm, 190 nm, and 40 nm for solutions A, B, and D, respectively. The QD thin film is the collection of tiny individual QD islands when higher hexane is used (Solution D). Droplet marks known as the boundaries of QD islands are shown by the OM and 3D images in Fig. 3d, which shows a low-density of QD particles. When using hexane as a solvent in the QD solution, the touch of QD droplets on the substrate forms the coffee-ring shape due to quickly evaporation. The diameter of 10 μm in the uniform QD droplets marks was reported^9^. The comparison of these results indicates the mixed-solvents influence in improving the surface structure.

**Figure 4:**
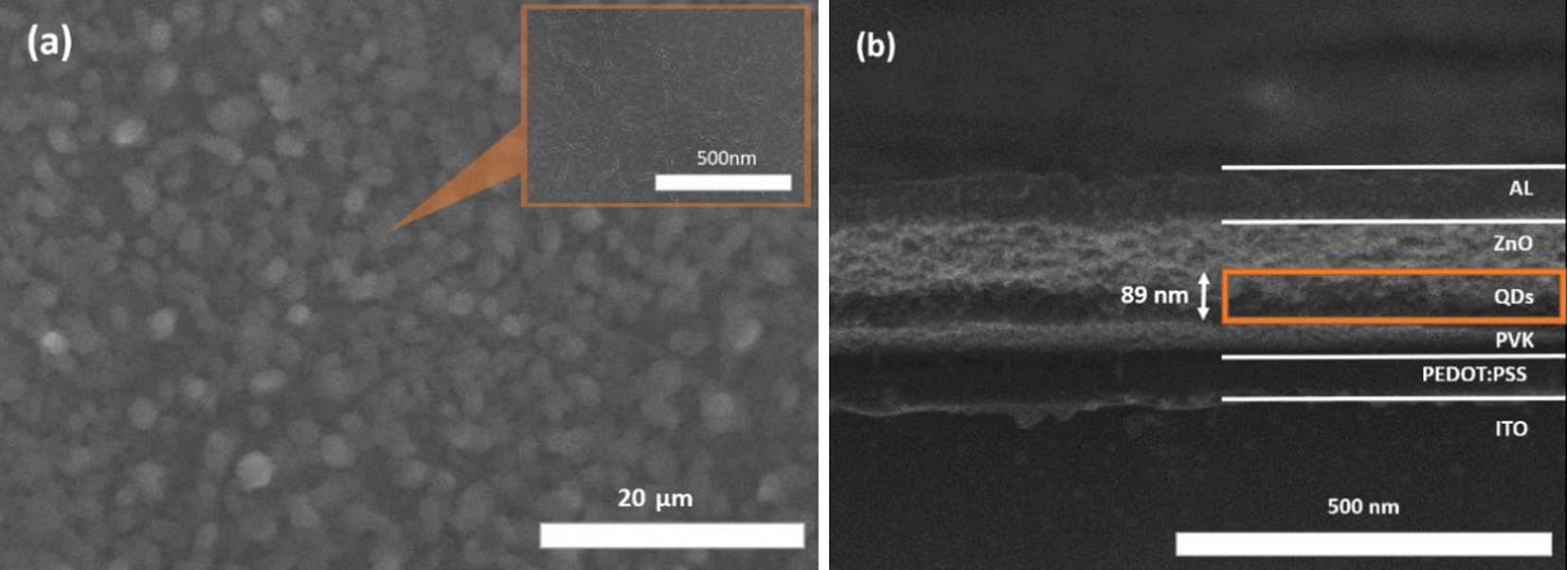
(**a**) SEM image of QD clusters of thin film after electrospray, and (b) cross-sectional SEM image of QD-LED device of ITO/PEDOT:PSS/PVK/QDs/ZnO/Al multi-structure.

Figure 4a shows SEM images of the QD layers prepared from Solution C on ITO glass with a deposition time of 15 min of continuous spraying (about 80–90 nm thickness). A continuous thin film is the collection of uniform QD cluster with 500 nm to 2 µm of diameter, which observed by the bright islands. The QD islands of the SEM images were well matched with the drop size previously calculated by the Scaling law. This figure also shows a very high density and uniformity of QD nanoparticle clusters.

The quality of electrosprayed QD films was confirmed by preparing QD-LED devices from different solvent mixtures with the structure of ITO/PEDOT:PSS/PVK/electrosprayed-QDs/ZnO/Al, as shown in Fig. S2 in the S.I. Figure 4b shows a cross-sectional SEM image of a QD-LED sample. This image clearly shows an electrosprayed QD layer of about 80 nm. Generally, an optimized QD-LED has high performance at an optimal thickness of about 100 nm for the PEDOT:PSS layer, 60–70 nm for the PVK layer, and 110–120 nm for the ZnO layer.

**Figure 5:**
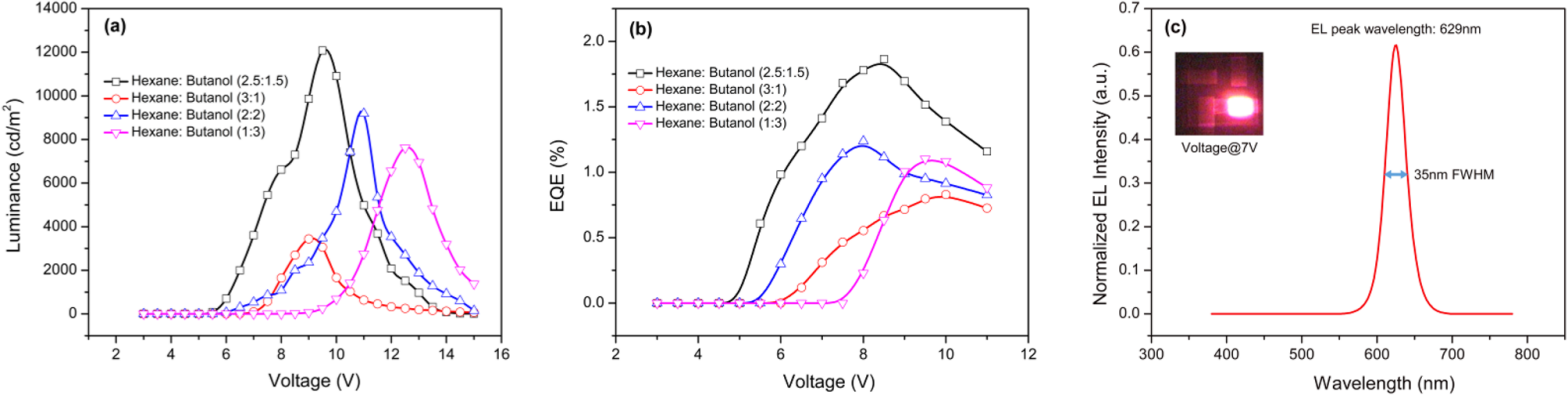
Optical characteristics (**a**) L-V and (**b**) EQE-V of the QD-LED devices using electrosprayed QDs with various solvent mixtures. (**c**) EL spectra and illuminating image of red QD-LED at 7 V (inset picture).

Figure 5a and b show the voltage-luminescence (V-L) and EQE of the QD-LED devices with four different volume ratios of the solvent. Because it is much less volatile than n-hexane, the addition of n-butanol reduces the rate of evaporation and helps to maintain a stable electrospray, resulting in smooth and uniform QD thin films. Solution C achieved a brighter and more efficient device with a maximum luminance of 12,082 cd m^−2^, the turn on voltage of 5.5 V, the maximum current efficiency of 3.93 cd A^−1^, and the highest EQE of 1.86%. The different volume ratios of the mixture can result in QD film that is too wet or too dry. A film that is too dry results in rough films with pinholes on the surface and affects the quality of the next coated layer. In contrast, a film that is too wet could cause damage to the HTL layer, resulting in lower brightness and current efficiency. Figure 5c presents the electroluminescence (EL) spectrum of the highest performance QD-LED device was recorded at the bias voltage of 7 V. It shows a characteristic red QD EL peak centered at ~ 629 nm with a full width at half maximum (FWHM) of ~ 35 nm. Very bright and uniform red emission can be observed by the inset picture.

## Conclusion

The Scaling law was adapted to verify the mechanism of EHD electrospray deposition process. Based on the electrospray mechanism, we calculated the initial QD droplet size, the effect of the rate solvent evaporation, and the prediction of the flight time of the electrospray. The results were in agreements with the experimental data obtained using the mixed solvent QD system. The layer by layer deposition of QD droplets shows a good surface roughness of 0.0308 µm. The QD-LED demonstrates a maximum luminance of 12,082 cd m^−2^, a maximum EQE of 1.86%, and a maximum current efficiency of nearly 4.0 cd A^−1^. This EHD electrospray method shows advantages such as straightforward and flexible control, low cost, high material utilization, large-scale processability, and compatibility with multi-structure devices like QD-LEDs and all-printed electronic devices.

## Methods

### Preparation of materials

A solution precursor was prepared by the dilution of 1 ml of red CdSe/CdS/ZnS colloidal QDs (Ecolumy Co., Ltd.) with various volume ratios of n-hexane and n-butanol to achieve a concentration of 10 mg/ml, followed by stirring at 300 rpm for 60 min.

To form an electron transport layer (ETL), ZnO nanoparticles were synthesized according to a previous report^19^. In brief, 1.18 g of zinc acetate dehydrate and 50 ml of methanol were added into a three-neck flask and stirred at 65 °C. A solution of 0.592 g of KOH in 26 ml of methanol was then dropped into the flask at 65 °C over a period of 15 min. The reaction mixture was stirred for 2.5 h vigorously at 1,000 rpm. After cooling to room temperature, the supernatant was decanted, and the precipitate was washed three times with 20 ml of methanol using a centrifuge to obtain ZnO nanoparticles. Next, 28 ml of *n*-Butanol, 2 ml of methanol, and 2 ml of chloroform were added to disperse the precipitate and produce a ZnO nanoparticle solution with a concentration of 6 mg/ml. The ZnO nanoparticle solution was filtered through a 0.45-µm PTFE-H syringe filter^20^.

### Preparation of EHD electrospray process

A diagram of the EHD electrospray coating process is shown in Fig. 1a. The equipment consists of several major components: a syringe pump, a syringe, a metal needle serving as a nozzle, a high voltage source, and a motorized substrate serving as a collector. The entire process was interfaced with a computer and monitored with a high-speed camera.

The QD solution was ejected through a metallic nozzle (I.D. 0.10 mm; O.D. 0.23 mm) using a micro-pump. The spraying speed was controlled with the system to deliver the ink, syringe pump, and nozzle. High voltage was then applied to induce an electric field between the nozzle tip and the substrate. The electric power was typically kept at 10 kV, and the distance between the metal tip of the nozzle and substrate was maintained at 6.5 cm. The low flow rate was typically maintained at 0.016 µL/s, and the spraying time was changed in the range of 10–15 min to identify a suitable thickness of QD thin films for various requirements.

The substrate was repeatedly moved at 2 mm/s along the diagonal of the ITO substrate (Fig. 1b). The control of the moving holder substrate is one of the critical factors in the QD thin film formation when using the EHD electrospray printing system. This factor guarantees that the QD particles cover all of the surface and that the thickness of the patterns is the same in every position. Moving the holder substrate along the diagonal also helps to use less materials (Fig. 1c).

### Fabrication of QD-LED devices

For the device investigation, QD-LEDs were fabricated and tested using a conventional structure of ITO/PEDOT: PSS/PVK/QDs/ZnO/AL/UV-epoxy/glass-encapsulation layer (Fig. S2 in the S.I.). The ITO-patterned glass (20 Ω/□) was first cleaned with deionized water, isopropyl alcohol, and acetone under ultra-sonication for 20 min and then exposed to UV-zone irradiation for 20 min. A PEDOT:PSS (Clevios P VP AI 4,083) layer was formed by spin coating as a hole injection layer (HIL) at 3,000 rpm for 60 s, followed by annealing at 150 °C for 30 min in ambient air. Next, PVK solution was spin-coated as a hole transport layer (HTL) onto the PEDOT:PSS layer at 3,500 rpm for 60 s and annealed at 150 °C for 30 min.

The QD layer was then deposited by the EHD electrospray printing system within 10–15 min, followed by the spin-coating of synthesized ZnO nanoparticles at 2,500 rpm onto the EML layer, and baking at 110 °C for 30 min. The top Al cathode was then deposited with an active layer device area of 4 mm^2^ by thermal evaporation. Finally, encapsulation was performed using UV-curable epoxy and treated by a UV source for 15–20 s with a thin glass cover^20^.

The surface morphology of the QDs was observed by an optical microscope, SEM (Hitachi S-4700), and NanoSystems NV- 2000. EL spectra and current density–voltage–luminance (J–V–L) characteristics were obtained using a SpectraScan PR 670 Spectroradiometer coupled with a Keithley 2,400 source measurement unit. All measurements were recorded under ambient conditions.

## Supplementary information

Supplementary file1
