# Supplementary material for: Electrospray mechanism for quantum dot thin-film formation using an electrohydrodynamic jet and light-emitting device application
Source: Sci Rep. 2020 Jul 6;10:11075. doi: 10.1038/s41598-020-67867-w (PMC7338508; doi:10.1038/s41598-020-67867-w)
Supplement: Supplementary file 1 — Supplementary file1 [file 41598_2020_67867_MOESM1_ESM.docx]

**Supplementary Information**

**Electrospray mechanism for quantum dot thin-film formation using electrohydrodynamic jet and light-emitting device application**

Tuan Canh Nguyen and Woon-Seop Choi*

School of Electronics and Display Engineering, Hoseo University, Asan, Chungnam 31499, Korea

*E-mail: wschoi@hoseo.edu


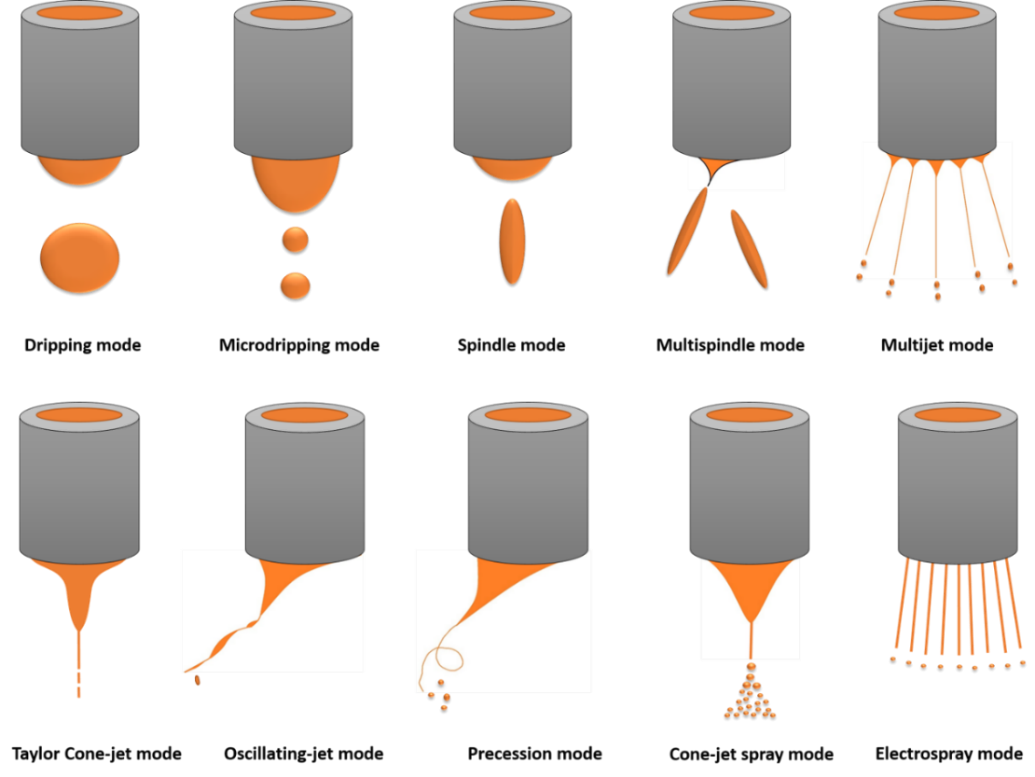


**Fig. S1.** Various printing modes of the EHD jet printing.

**
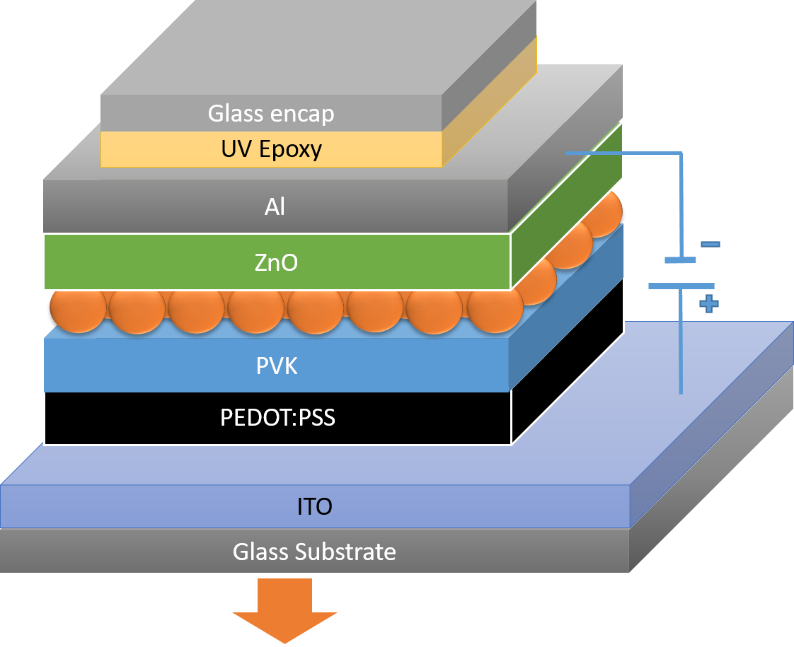
**

**Fig. S2**. A QD-LED device with multi-structure of ITO/PEDOT:PSS/PVK/electrospray QDs/ZnO/Al/UV epoxy-glass encap.


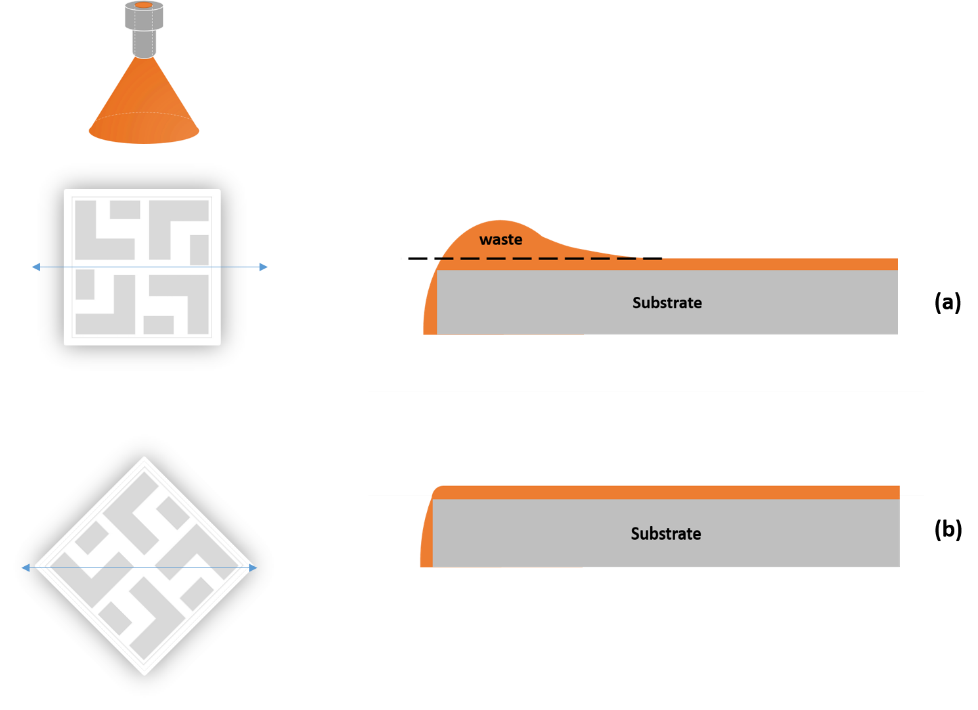


**Fig. S3** Spray shape of QD films with (a) the side and (b) the diagonal direction of the substrate.

**Parameter calculations of electrospray mechanism in EHD jet**

**Eq 1. Scaling law equation^1^:**

$D_{d}$*=*$G(\kappa)({\frac{Q\kappa\varepsilon_{0}}{K})}^{\frac{1}{3}}$

*G(*$\kappa$*) =10.87*$\kappa^{{-6}/5}$ *+ 4.08*$\kappa^{{-1}/3}$

$D_{d}$ is the droplet diameter (µm), $\kappa$ is the dielectric constant, Q is the flow rate, $\varepsilon_{0}$ is the permittivity of vacuum, and K is the liquid electrical conductivity (µS/cm)

*ε*_0_  permittivity of vacuum 8.85×10^−12^ F⋅m^−1^

Electrical conductivity hexane: 0.04 pS /m

Electrical conductivity butanol: 9.12 pS /m

Dielectric constant butanol: 17.7 (20^o^C)

Dielectric constant hexane: 1.88 (25^o^C)

Dielectric constant quantum dots CdSe/ZnS: 8.85 (25^o^C) (Cadmium Selenide/Zinc Sulfide)

The flow rate of solution was set as 0.016 µl/ sec

Because of this particularly mode structure, it is difficult to clarify how many jets in this plume of QD droplet. Therefore, to apply Scaling law and predict the diameter of initial QD droplets, we assume that the flow is indeed so divided among the number of jets, then the average flow rate per jet will be calculated by $\left. \left( \frac{Q}{n} \right. \right)^{1-\alpha}$. In which: Q is the initial flow rate, n is the number of jets, α is coefficient. In case of the number of jets is small, we can predict the size of QD droplet by simulation, image analysis, or applying by the Groove model.

In spite of large number of jets in this research, we assume that there are hundred jets (n=100) and α=0 for simplification, then the average flow rate per jet will decrease 100 times.^5^ Therefore, the estimated diameter of the initial QD droplets (0.6 µm to 3.169 µm) will reduce ∛100 to (0.13 µm to 0.68 µm). This estimated value completely matches with the averages of QD islands shown by the cluster on the SEM top-view image (500nm -2µm).

**Eq. 2. The complete evaporation time for a droplet can be calculated by the following equation^2^:**

$$\tau_{e}\tau_{e}=\frac{R\rho_{d}T_{d}D_{d}^{2}}{8D_{g}MP_{d}exp\left( \frac{4\sigma\nu_{m}}{D_{d}RT_{d}} \right)}$$

Where R is the universal gas constant, ρd is the density of the droplet, $T_{d}$ is the droplet temperature (all the electrospray experiments were conducted at 25 °C), $D_{d}$ is the diameter of the droplet, $D_{g}$ is the diffusion coefficient of the solvent, M is the molecular weight of the solvent, Pd is the partial pressure of the solvent, and ϭ and νm are the surface tension and molar volume of the liquid, respectively

- Evaporation time determines the time of coating and the speed of moving (2mm/sec).

**Eq.3. The fight time of electrospray droplets between the needle and the collector^1,3^:**

$$\tau_{res}=\frac{18\mu Qd^{2}}{ICD_{d}^{2}\triangle V}$$

Where d is the collection distance (6 cm), $\triangle V$ is the applied voltage (10 kV), I is the droplet current, $\mu$ is the viscosity of the fluid, Q is the flow rate 0.016 µl/ sec, C is Cunningham slip correction factor, and $D_{d}^{2}$ is the diameter of the droplet. We estimated the flight time is from 0.23 to 0.85 ms.

Calculation process of the current I is as following^1,4^:

$I=f(\kappa)\left( \frac{\gamma KQ}{\kappa} \right)^{1/2}$ (1)

$f\left( \kappa\right)= -449-0.21\kappa+157\kappa^{1/6}+336k^{-1/6}$ (2)

Where: I is the droplet current generated by charged droplet in the electric field, γ is the liquid surface tension, $K$ is liquid electrical conductivity, $\kappa$ is dielectric constant, and Q is feed flow rate.

The calculation process of Cunningham slip correction factor (C) is as following^4^:

$C=1+\frac{2\lambda}{D_{d}}(1.257+0.4\exp\left( \frac{-1.1D_{d}}{2\lambda} \right))$ (3)

Where $\lambda$ is mean free path of air, $D_{d}$ is the droplet diameter, and $D_{d}$ is the droplet diameter.

- Estimated flight time from 0.23 to 0.85 ms is less than the interleaved time of coating (stopping time between two turns moving the substrate)
- The calculated parameters are well matched with experimental results.

**References**

[1] Chen, D. R., Pui, D.Y.H. Pui, *Aerosol Sci. Technol.*, **27,** 367-380 (1997).

[2] Shah, V. B., Biswas, P. *ACS Nano*, **8**, 1429–1438 (2014).

[3] Ju, J., Yamagata, Y., Higuchi, T. *Adv. Mater****.*** **21**, 4343–4347 (2009).

[4] Kavadiya, S., Raliya, R., Schrock, M., Biswas, P., *J. Nanopart. Res.,* **19**, 43 (2017).

[5] Ryan, C. N., Smith, K. L. & Stark, J. P. W. *J. Aerosol Sci.*, **51.** 35-48 (2012).
